# Circumcision registry promotes precise research and fosters informed parental decisions

**DOI:** 10.1186/s12910-018-0337-7

**Published:** 2019-01-09

**Authors:** Robert S. Van Howe, Morten Frisch, Peter W. Adler, J. Steven Svoboda

**Affiliations:** 1 Department of Paediatrics, Central Michigan University College, 413 E. Ohio Street, Marquette, MI 49855 USA; 2 Department of Clinical Medicine, Center for Sexology Research, Aalborg University, DK-9000 Aalborg, Denmark; 3 University of Massachusetts (Lowell), Lowell, MA USA; 4 Attorneys for the Rights of the Child, Berkeley, CA USA

## Abstract

**Background:**

In 2017 Ploug and Holm argued that anonymizing individuals in the Danish circumcision registry was insufficient to protect these individuals from what they regard as the potential harms of being in the registry (overreaching social pressure, stigmatization, medicalization of a religious practice, discrimination and promoting polarized research).

**Discussion:**

We argue that Ploug and Holm’s fears in each of the areas are misguided, not supported by the evidence, and could interfere with the gathering of accurate data. The extent of the risks and harms associated with ritual circumcision is not well known. The anonymized personal health data supplemented with the circumcision registry will enable more precise research into the medical consequences of ritual circumcision, and allow parents to make more fully informed decisions about circumcision with minimal, if any, adverse consequences.

We read with interest the recent publication by Ploug and Holm regarding the Danish circumcision registry [1]. As they note, the main purpose of the registry is to enable future research into the consequences of circumcision, and they concede that research into personal health data holds great potential for improved treatment. They argue, nonetheless, that anonymizing the males listed in the registry is insufficient to protect these individuals from the potential harms of being in the registry, and they ask whether informed consent should be obtained before inclusion in the registry. Even though they acknowledge that the potential for harm is speculative, the authors identify five sources of concern: 1) overreaching social pressure; 2) group stigmatization; 3) medicalization of a religious practice; 4) group discrimination; and 5) the potential bias in circumcision research as a polarized field of study. We argue that their fears are misguided and not supported by the evidence.

## Overreaching social pressure

Ploug and Holm argue that a circumcision registry may be perceived by adults who favour circumcising boys for religious reasons as a form of social pressure that threatens their self-expression and self-development, and that the registry, “may infringe [their] self-determination and it may cause [them] harm.” The authors also argue that social pressure may denormalize the practice of male circumcision. These arguments are not convincing.

First, regarding the concern that social pressure may *denormalize*, the practice of male circumcision is already denormalized. That is, circumcision of male children has been practiced in some geographies and cultures for thousands of years, it has on a wider scale been the exception and not the norm. Second, it is improbable that anyone who has a genuine and deep religious commitment to circumcise his or her son and learns of the establishment of an anonymous circumcision registry will abandon the practice because of it [2].

Third, we all have an interest in accurate public health research and of knowing to what extent any medical procedure — in this case the removal of the foreskin of the penis — risks harming or does harm boys and the men they become. Whether performed in a sterile hospital setting or otherwise, non-therapeutic circumcision is an invasive surgical intervention. As such, it entails the risks of any invasive procedure, along with the specific risks associated with the removal of the foreskin, including a significant risk of meatal stenosis, [3] serious disfigurement, [4] hemorrhage, infection, [5] and on rare occasions, death [6]. Even when there are no “complications,” circumcision is itself a physical injury as it removes functional tissue with known erogenous, protective, and immunological properties; it can also cause psychological harm [7–9].

Insofar as parents choosing to circumcise their children risk significantly attenuating the health of their sons, it is important to gather accurate information regarding the harms of circumcision so that parents who make this decision on behalf of their children can do so in an informed fashion. In this sense, the gathering of health statistics increases the capacity for religious adults and/or communities to express their agency and autonomy: a fully-informed decision is more autonomous than one that is unexamined or based on wishful thinking. Whether information regarding the harms associated with the practice is seen by parents as grounds to abandon the practice is up to the parents; the registry is a vehicle for providing accurate information. What people do with this information is up to them. An incomplete registry would result in incomplete information, which can lead to uninformed decisions, and hence less autonomous decision-making.

Fourth, male genital cutting is usually performed for religious, cultural, and personal reasons, and not for medical reasons [10]. While Ploug and Holm do not take an overt position on the ethical permissibility of circumcision, the tenuous ethical position of the practice highlights the need to bring the most accurate empirical evidence possible into the debate to minimize the potential for polarization discussed below. While Ploug and Holm express concern regarding the social pressures surrounding circumcision, it must be noted that circumcision is itself an extreme form of social pressure that does not merely threaten the self-development of boys and the men they become — it irreversibly changes an intimate part of their bodies without their consent, removing a part of their genitals with which adult men rarely volunteer to part. In other words, Ploug and Holm appear to confuse the interests that society extends to the individual with the interests of the group. Pre-enlightenment thinking viewed children as a possession of their family and their community. Contemporary society considers the individual, even a child, to be of primary ethical importance [11]. Accordingly, legal scholars argue that children, including boys, are themselves entitled to protection form certain group-level social pressures in virtue of their own right to freedom of religion, i.e., their right to choose to affiliate with any religion or no religion at an age of understanding. Such affiliation may be compromised by having previously been permanently and involuntarily marked as a member of their parents’ religion [12]. One must ask, then, whether the child in this example, who is powerless to resist a community-enforced encroachment upon his body, is at greater risk of harm from being circumcised than the community is by virtue of having health statistics recorded about its invasive rites. In order to answer this question – in addition to conceptual, moral, and legal analysis – the most accurate empirical evidence concerning the effects of the practice must also be brought to bear [13]. A circumcision registry does not interfere with that aim, but rather supports it.

## Group stigmatization

The authors note that the specific religion of boys “can easily be deduced from the timing of circumcision, the name of the boy, and immigration status of the parents.” This demographic information, which is easily available, is already sufficient to ground stigmatization, if that is the worry. There is no evidence that a circumcision registry would contribute anything further. If one is aware that a Danish male is Jewish or Muslim, one can reasonably infer, to a certain extent, that he is circumcised. It is unfounded speculation to assume that the circumcision registry would add to any such stigmatization if it does exist.

## Medicalization of a religious practice

In their discussion of the fear that religious circumcision will become medicalized, the authors repeatedly refer to “everyday activities.” Cutting the genitals of a minor is not an “everyday activity.” While babies are born every day, for an individual mother/baby pair, the birth of the baby is not an “everyday” event. If one is to consider circumcision to be a profoundly important religious/cultural event, one should not place it in the same category as mundane, everyday activities. It is a special event. There is no evidence that a circumcision registry would change this.

Circumcision has medical aspects to it, so some medicalization is inevitable. It involves cutting and removing tissue. As stated, it can result in complications such as bleeding, infection and, occasionally, death. Given the complications associated with the practice, one could argue on the basis of ensuring public safety that only licensed medical practitioners be allowed to perform circumcisions.

The authors express fear that the medical aspects of circumcision will replace the religious aspects as the primary focus of the event. Evidence from the United States suggests that this is unlikely to occur. Circumcision was introduced there as a medical procedure in the Nineteenth Century, but today is performed primarily for cultural rather than medical reasons [10]. Consequently, the authors’ fears of medicalization are overstated and unlikely to take place.

One of the duties that we expect of the medical establishment and of the state is to protect the most vulnerable from harm. The authors make a bare assertion that protecting a child from the harm of having his genitals cut without his permission is beyond the purview of the medical establishment or the state, Ploug and Holm do not even attempt to demonstrate why circumcision should be exempt from functions normally performed by medical and governmental authorities to protect children.

The medical aspects of circumcision are an important part of the decision-making process in religiously motivated circumcisions. The practice is not risk free. Certain sexual functions, including all those that involve manipulation of the foreskin itself, are of necessity lost; and others are placed at an increased risk of harm. In the end, the physical losses need to be assessed against the putative religious/cultural gains.

To summarize, it is inescapable that circumcision has a medical aspect to it. Nevertheless, it seems unlikely that medical concerns will replace religious/cultural enticements as the primary motivation to continue the practice.

## Group discrimination

The authors are concerned that the circumcision registry singles out a minority cultural practice for monitoring. Potentially harmful practices are identified on a regular basis to determine their impact on health and fundamental well-being. The authors provide the example that sports activities are not monitored, but there are two problems with this example. First, sports are engaged in with the age-appropriate consent and involvement of the child: the nature of the activity and the type of risk to which he is exposed through his participation differ markedly from the nature and risks of circumcision. In the latter case, he is a passive participant, and has no ability to determine whether the risks are worth it to him because of the gains he expects to achieve through his involvement. Second, perhaps sports should be monitored with a registry. Football in the United States, for example, is coming under closer scrutiny to determine the level of exposure needed to result in chronic traumatic encephalopathy and it is likely that registries are on the horizon. Any discrimination from a circumcision registry would be incidental and the registry would only provide more precise information than is already available from the demographics associated with the genital cutting of minor males. Using the authors’ logic, it would be discriminatory to have a registry of patients with sickle cell anemia because the illness affects primarily people of relatively recent African heritage. If the object of interest happens to occur primarily within a demographic minority, is study of that object discriminatory? Without further premises added to the argument, the answer is no. In fact, insofar as society monitors the health of boys and men in various ways, it would discriminate against boys and men in groups practicing circumcision not to monitor the effect of circumcision on their health.

## Polarized research

Male circumcision is an extremely divisive topic to the degree that it is difficult to determine whether an expert’s views are based on the findings of legitimately performed research or whether the findings of research are driven by the researcher’s pre-existing views. All researchers in all topics have an expectation bias. They would not have the energy to pursue an experiment or study if they did not have a specific outcome in mind. Hypothesis generation is an integral part of the scientific process. Eliminating or weakening the circumcision registry does not address this issue. It is up to the entity that controls the information in the registry to determine whether the data will be used in a scientific and ethical manner. In this instance, the data are kept by a national entity that does not have financial or other potential conflicts of interest. Moreover, to minimize the risk for unqualified or biased research, data will only be released for use by qualified researchers whose projects have been approved by the national Data Protection Board. If what the authors are arguing for were adopted, then no national registry could exist on any demographic or medical factor.

The answer to polarization is not to encourage incomplete data collection. The answer to polarization is to collect the most accurate and complete data possible. It is the very absence of high-quality data that often allows polarized researchers to veer off into their respective corners, as they “fill in the gaps” in evidence with their own preferred inferences and biases.

## Anonymized data

Is using anonymized data enough protection? Contrary to what the authors claim, no one believes that using anonymized data provides complete protection. Still, the authors show very little trust in the ability of research ethics committees and data protection boards to protect the individual. They argue, without providing evidence in support, that these committees and boards may take on the “objective view” of the “equanimeous[sic] Danish citizen” rather than the “subjective view” of the young Muslim man.

The authors provide a special pleading for the case of circumcision in minors. Anonymizing data, in general, has worked well so far, and nothing the authors present indicates the need to change course. Without impeccable data, polarization will only increase. If, as the authors suggest, we need to analyze database-based research on a case-by-case basis, then there would be a roadblock to any research that uses a national database. Requiring informed consent, even meta-consent, presents enough of a barrier to prevent research from being undertaken [14]. Given the important findings extracted from national databases, such impediments would hold back important discoveries.

## Conclusion

Ritual circumcision entails risks and harms, but the extent of the risks and harms is not well known. The anonymized personal health data supplemented with the circumcision registry will enable more precise research into the medical consequences of ritual circumcision, and allow parents to make more fully informed decisions about circumcision with minimal, if any, adverse sequelae. The concerns raised by Ploug and Holm are unfounded and spurious. Their recommendations are unnecessary to protect identifying information and would purposelessly interfere with data gathering that may help identify and eventually reduce the harms associated with circumcision.
