# Supplementary material for: Circumcision registry promotes precise research and fosters informed parental decisions
Source: BMC Med Ethics. 2019 Jan 9;20:6. doi: 10.1186/s12910-018-0337-7 (PMC6327488; doi:10.1186/s12910-018-0337-7)
Supplement: Related texts

# Reply: Response to “Circumcision Registry Promotes Precise Research and Fosters Informed Parental Decisions”

**Authors:** Thomas Ploug, Søren Holm

**Affiliations:** 5 0000 0001 0742 471X grid.5117.2 Centre for Applied Ethics and Philosophy of Science, Department of Communication, Aalborg University Copenhagen, A C Meyers Vænge, 2450 København SV, Denmark; 6 0000000121662407 grid.5379.8 University of Manchester, Centre for Social Ethics and Policy, School of Law, Manchester, M13 9PL UK; 7 Center for Medical Ethics, Faculty of Medicine, University of Oslo, Oslo, Norway; 8 0000 0001 0742 471X grid.5117.2 Centre for Applied Ethics, Aalborg University, Aalborg, Denmark

## Response

We thank Howe et al. for the response to our paper. As prominent anti-circumcision activists and academics it is perhaps understandable that they seem to read our paper as an intervention in the debate about male circumcision, but that is a misreading, as we already state clearly in our paper. Their response ignores our stated arguments and instead argues against straw men. We will therefore commence with making it entirely clear what is argued.

We argued that anonymised registry-based research may be associated with types of harm that individuals may have an interest in avoiding, and for that reason individuals should be recognised as having a right to protect themselves against these harms through the provision or refusal of consent to participate in such research [1].

Essentially, we argued that registry-based research is similar to other kinds of research where there may be great benefits for society and individuals, but where there are also risks of harm. In all such cases the individual is recognised as having a right to weigh benefits and harms against each other and on this basis to provide or refuse consent. As we also point out, repeatedly, that right is not absolute. It can be outweighed by other considerations, but that does not diminish its status as a right.

We used the case of the Danish Circumcision Registry to illustrate the respective harms. However, our argument has nothing to do with the circumcision debate per se. And, contrary to the authors of the response, we do not hold any particular views on the legitimacy of male circumcision in minors or adults. Their claim that “the authors provide a special pleading for the case of circumcision in minors” demonstrates an unwillingness to engage in the actual debate in our paper, namely the issue of informed consent for anonymous registry-based research.

In light of this it is perhaps not surprising how much effort is devoted to listing the harms of circumcision throughout the response. However, even if such harms are recognised they are at most reasons against circumcision. They are not reasons for coercing individuals into registry-based research any more than the prospect of a cure of prostate cancer is a reason for coercing individuals to participate in such research. Most other medical research is aimed at producing real and significant health care benefits for individuals, but such potential does not in itself negate the requirement of informed consent, and especially not in cases where the individual is at risk of suffering harm.

Hence, in order to counter our argument the authors must show both 1) that mandatory participation is necessary, and 2) that the suggested harms are not real.

We contend that van Howe et al’s claims over-emphasise the importance of having accurate and precise information about circumcised boys and men, and especially over-emphasise a further claim that this implies having complete information in the registries used for research. Thus participation in registry-based research such as the circumcision registry should be mandatory because otherwise information will be incomplete, and this will lead to biased results about the effects of circumcision. This argument is flawed for three reasons. First, it tacitly assumes that completeness cannot be achieved voluntarily. There is significant evidence to the contrary [15]. Second, there is no straightforward logical connection between completeness and accuracy. Accuracy may be achieved on the basis of incomplete data. Third, most registries are incomplete to some degree and this does not seem to have posed an obstacle to epidemiological research presently or in the past [16]. Hence, the authors fail to establish 1) above, namely that mandatory participation is necessary. But what about 2) – do the authors succeed in showing that he harms are not real?

The authors claim that social pressure cannot lead to the denormalization of circumcision because it is already denormalized. However, their conception of what it means that a social practice is denormalized is puzzling. They write:

“…the practice of male circumcision is already denormalized. That is, circumcision of male children has been practiced in some geographies and cultures for thousands of years, it has on a wider scale been the exception and not the norm.”

But that claim entails both that circumcision is not normal, but already denormalized in Saudi Arabia where more than 95% of the population are Muslims and circumcise their sons; and that social practices like strict respect for human rights are already denormalized in Denmark and the UK, because they are “on a wider scale the exception”. But that cannot be right. Male circumcision is normal in Saudi Arabia and respect for human rights normal in Denmark and the UK, whether or not it these practices are ‘normal’ world-wide. What is conceived as normal in a given society, and what can therefore be denormalized is influenced by what is seen as normal in other societies, but it is not definitionally or analytically tied to any conception of a global ‘normal’.

In terms of stigmatization and medicalization, the authors deny that a circumcision registry could add anything to stigmatization of religious minorities and the medicalisation of these practices, and they think that we should provide evidence to show that it does. However, the burden of proof in relation to the potential harms of conducting a certain type of research will always fall to the researchers. We have provided several reasons for believing that stigmatization and medicalisation is likely to follow; and there is evidence that stigmatization may have a number of negative effects including lowered self-esteem, anxiety and depression [17, 18]. The account of medicalization the authors rely on is again quite odd, and differs markedly from the standard account in the literature. They write: “Circumcision has medical aspects to it, so some medicalization is inevitable.” However, all human activities have ‘medical aspects to them’, but they are not all medicalized. What the concept of medicalization points to is that it matters in a number of different ways whether a given practice or activity comes to be understood in medical terms and inscribed in a medical framework of meaning and value [19].

The response also has a number of problems in relation to our analysis of discrimination. First, our claim is misrepresented. They state:

“Let us define discrimination as involving acts and practices that confer harm upon a group of people by treating them differently from relevantly similar groups in society. The very existence of the circumcision registry may be claimed to be discriminatory in this sense because 1) it singles out a minority cultural practice for monitoring of and research into the associated risks among a number of practices associated with relevantly similar risks, and 2) the singling out of this practice may confer the harms of stigmatization and medicalization on group members.”

Let us first note, pace the implicit assertion in the response that incidental harm is also harm and should be taken as seriously as any other harm. Causing intentional harm may be more blameworthy than causing incidental harm, but the person who is harmed incidentally is harmed to the same extent as the person who is harmed intentionally. It is therefore irrelevant whether harm caused by discriminatory effects of a particular registry is caused incidentally or intentionally. But, perhaps more important is that the operative conception of discrimination here is *causing harm by differential treatment*. It therefore does not follow from our analysis that:

“… it would be discriminatory to have a registry of patients with sickle cell anemia because the illness affects primarily people of relatively recent African heritage.”

On our analysis, whether a sickle cell anemia registry will be discriminatory will depend on whether it causes harm, incidentally or intentionally to ‘people of relatively recent African heritage’. And the answer to that question will clearly depend on exactly in which society we envisage the establishment of such a register. Such a register may be innocuous in Denmark, but as the literature on sickle cell disease and racism in the USA shows it is not obvious that it would be purely beneficial in that country [20, 21].

Second the response presents the point that “… perhaps sports should be monitored” as a criticism of our paper, but we explicitly raise this point ourselves:

“The inequitability point does, however, cut both ways because it can also be claimed that those participating in these other activities [sports] have a claim that their problems should be investigated.”

Second, let us turn to the argument that the use of data can be adequately regulated by data protection agencies and research ethics committees. As we have explained in our paper, the only relevant agency in Denmark is the Danish Data Protection Agency. In this context it is interesting to briefly look at the largest project using Danish registry data to study the effects of childhood male circumcision [22]. This study was approved by the Danish Data Protection Agency based on application 2009-41-4154, but the word circumcision does not occur anywhere in the application that was approved. The Agency therefore had no possibility to even consider whether the project was contentious or not before approving the use of data. Van Howe et al. do not recognise this. Its omission significantly weakens their claims about the adequacy of the protective functions of research ethics committees and data protection agencies.

We should perhaps note that there is a very fundamental agreement between us, and the authors of the response. They write:

“Contemporary society considers the individual, even a child, to be of primary ethical importance.”

We completely agree that the individual is of primary ethical importance. But, if that is the case it is not only an ethnic or religious group that cannot override that importance without sufficient justification, it also applies to medical researchers and society. As a matter of ethics it is not (as claimed by the authors) society that extends interests to the individual, it is the individual who has rights and interests that contemporary societies recognise.
